# Unexpected Fat Distribution in Adolescents With Narcolepsy

**DOI:** 10.3389/fendo.2018.00728

**Published:** 2018-12-06

**Authors:** Natasha Morales Drissi, Thobias Romu, Anne-Marie Landtblom, Attilla Szakács, Tove Hallböök, Niklas Darin, Magnus Borga, Olof Dahlqvist Leinhard, Maria Engström

**Affiliations:** ^1^Department of Medical and Health Sciences (IMH), Linköping University, Linköping, Sweden; ^2^Center for Medical Image Science and Visualization, Linköping University, Linköping, Sweden; ^3^AMRA Medical AB, Linköping, Sweden; ^4^Department of Clinical and Experimental Medicine (IKE), Linköping University, Linköping, Sweden; ^5^Department of Neuroscience, Uppsala University, Uppsala, Sweden; ^6^Department of Pediatrics, Institute of Clinical Sciences, Sahlgrenska Academy, University of Gothenburg, Gothenburg, Sweden; ^7^Department of Biomedical Engineering (IMT), Linköping University, Linköping, Sweden

**Keywords:** orexin, hypocretin, brown adipose tissue, visceral adipose tissue, subcutaneous adipose tissue, BMI, magnetic resonance imaging (MRI), obesity

## Abstract

Narcolepsy type 1 is a chronic sleep disorder with significantly higher BMI reported in more than 50% of adolescent patients, putting them at a higher risk for metabolic syndrome in adulthood. Although well-documented, the body fat distribution and mechanisms behind weight gain in narcolepsy are still not fully understood but may be related to the loss of orexin associated with the disease. Orexin has been linked to the regulation of brown adipose tissue (BAT), a metabolically active fat involved in energy homeostasis. Previous studies have used BMI and waist circumference to characterize adipose tissue increases in narcolepsy but none have investigated its specific distribution. Here, we examine adipose tissue distribution in 19 adolescent patients with narcolepsy type 1 and compare them to 17 of their healthy peers using full body magnetic resonance imaging (MRI). In line with previous findings we saw that the narcolepsy patients had more overall fat than the healthy controls, but contrary to our expectations there were no group differences in supraclavicular BAT, suggesting that orexin may have no effect at all on BAT, at least under thermoneutral conditions. Also, in line with previous reports, we observed that patients had more total abdominal adipose tissue (TAAT), however, we found that they had a lower ratio between visceral adipose tissue (VAT) and TAAT indicating a relative increase of subcutaneous abdominal adipose tissue (ASAT). This relationship between VAT and ASAT has been associated with a lower risk for metabolic disease. We conclude that while weight gain in adolescents with narcolepsy matches that of central obesity, the lower VAT ratio may suggest a lower risk of developing metabolic disease.

## Introduction

Narcolepsy type 1 is a chronic sleep disorder, characterized by excessive daytime sleepiness (EDS) with frequent uncontrollable sleep attacks, cataplexy; a sudden loss of muscle tone often related to the experience of strong emotions, and other sleep related symptoms (1, 2). Furthermore, narcolepsy has been associated with a higher BMI (3). A study from 2013 investigating the prevalence of obesity among pediatric narcolepsy patients found that obesity affects more than 50% of children with narcolepsy and was also shown to be related to symptom severity (4). Similarly, rapid weight gain, and significantly higher BMI was reported in a study on pediatric narcolepsy patients following the H1N1 vaccination in Sweden (5, 6). High BMI in childhood is a known risk factor for metabolic syndrome and diabetes type 2 in adulthood (7, 8) and studies have also demonstrated an increased risk in narcolepsy patients (9, 10). Although BMI increase at narcolepsy onset is well-documented, the associated body fat distribution and the mechanisms behind weight gain in narcolepsy have still not been systematically investigated in humans. However, it has been suggested that loss of orexin producing neurons in the lateral hypothalamus may be involved (11).

Orexin is involved in the stabilization of sleep-wake cycles (12–14), and loss of this hormone causes narcolepsy in animal models (15–17). Besides being involved in sleep regulation, there are also widespread orexinergic projections to areas regulating energy homeostasis and feeding (18, 19). Additionally in animal models a loss of orexin leads to obesity even in the face of hypophagia (11, 20) indicating a specific role for orexin in regulating body adiposity. In humans evidence for hypophagia is more conflicting and has only been reported by a few investigators in narcolepsy patients with cataplexy (21, 22). Others report binge eating (23) or a significantly larger intake of carbohydrate rich snacks (24) to be associated with weight-gain in narcolepsy. A suggested mechanism for the involvement of orexin in the pathogenesis of obesity may lie in its actions on brown adipose tissue (BAT) (20, 25–27), a calorie-burning fat typically associated with newborns although its presence and metabolic activity has more recently also been verified in adulthood (28, 29).

This paper is published as part of a larger project aiming to reveal fundamental physiological mechanisms in narcolepsy (30, 31). The aim of this study was to investigate the body fat distribution in narcolepsy patients. Previous studies on weight gain in narcolepsy have used external measures such as BMI, waist-, or thigh- circumference to establish body fat profile though their accuracy has been challenged [reviewed in Freedman and Sherry (32)]. Instead, we used a new magnetic resonance imaging (MRI) method, which allowed us to specifically measure abdominal subcutaneous adipose tissue (ASAT), visceral adipose tissue (VAT), supraclavicular brown adipose tissue (sBAT) fat fraction, and muscle fat infiltration (MFI). These measures were also normalized to adjust for individual variations in height. Corresponding with previous evidence we expect to find differences in body fat distribution between our adolescent narcolepsy patients and their healthy peers. Furthermore, in consideration of the involvement of orexin in narcolepsy disease etiology and the reported evidence for its involvement in regulating BAT, we also expect to see differences in the fat fraction of sBAT, an indirect measure of BAT volume. To our knowledge, no previous study has investigated specific fat distribution in narcolepsy patients.

## Materials and Methods

### Patients

Twenty-one participants with narcolepsy type 1 were recruited from a population-based study in western Sweden (*n* = 15) (6) and from pediatric clinics in the county of Östergötland (*n* = 6). Diagnoses were based on the classification codes of the Swedish version of the International Classification of Diseases, Tenth Revision (ICD-10), and the diagnostic criteria for narcolepsy according to the 2005 International Classification of Sleep Disorders (33). Inclusion criteria for patients were a confirmed diagnosis of narcolepsy and being between 12 and 20 years of age at time of enrollment. Patients were excluded if there was evidence of cognitive disabilities.

Data from two narcolepsy patients were excluded from further analyses due to equipment malfunction (*n* = 2). This left a final sample size of 19 participants with narcolepsy (*N* = 8, females, *N* = 11, males). Average age of all patients was 16.3 years, *SD* = 2.2, and mean duration of narcolepsy was 4.8 years, *SD* = 1.2 (Table 1). For all but six patients, CSF orexin status (< 130 pg/ml) was taken at time of diagnosis whereof nine patients had orexin levels ≤ 10 pg/ml. Seventeen patients were being prescribed medication for narcolepsy (Methylphenidate, *N* = 15 or Modafinil, *N* = 2, and Fluoxetine, *N* = 8). Narcolepsy patients were allowed to take their prescribed medications prior to the exam. Data from narcolepsy patients were compared to 17 age and sex matched healthy controls, which were recruited by advertisement. Controls were confirmed to have no medical history of neuropathological diseases or mental illness by questionnaires and interviews prior to examination. The Regional ethical review board in Linköping, Sweden, approved the study (2013/99-31) and all participants gave informed consent to participate. For all participants under the age of 16, written informed consent was obtained from the parents.

**Table 1:** Demographic table.

**Patient**	**Gender**	**Age (years)**	**Narcolepsy duration (years)**	**CSF-orexin (pg/ml)**
1	F	17–21	6	81
2	M	17–21	5	< 10
3	M	12–16	4	< 10
4	M	17–21	5	Unknown
5	F	17–21	5	< 10
6	F	17–21	4	79
7	M	12–16	9	< 10
§8	M	17–21	5	< 10
9	M	17–21	4	49
10	F	17–21	5	Unknown
11	F	17–21	4	18
12	F	17–21	5	< 10
13	M	12–16	4	< 10
14	F	12–16	4	130
15	M	12–16	3	58
16	F	17–21	5	Unknown
17	M	12–16	4	Unknown
18	F	12–16	4	Unknown
§19	F	12–16	5	10
20	F	17–21	5	Unknown
21	F	17–21	5	< 10

*Narcolepsy patient demographics. §, excluded patients*.

### Actigraphy

All participants were monitored with actigraphy (Sense Wear, Body Media, Inc., Pittsburgh, PA, USA) using an armband with an actigraphy device mounted on the back of the left upper arm approximately 23 h each day during 1 week before the MRI examination. The actigraphy device contained a multisensory array including a 3-axis accelerometer, heat flux sensor, galvanic skin response sensor (GSR), and a skin temperature sensor. Algorithms taking these multisensory measures into account give estimates of e.g., total daily sleep and daily energy expenditure. Actigraphy data (Table 2) was used as covariates in the statistical analysis of adipose tissue, see section Statistics.

**Table 2:** Descriptive data.

	**Narcolepsy patients**	**Healthy controls**	**Statistics**
Sleep (h)	5.62 SD 1.72	6.73 SD 1.06	*p* = 0.028
Energy expenditure (J)	9568 SD 3120	10982 SD 2482	*p* = 0.145
Mean height (cm)	169 SD 11.7	172 SD 12.4	*p* = 0.462
BMI	24.72 SD 6.37	21.22 SD 2.42	*p* = 0.039
BMI_z_	0.80 SD 0.93	0.01 SD 0.67	*p* = 0.004

*Table shows descriptive data over narcolepsy patients and healthy controls obtained from the actigraphy, height, and weight measurements. Data of sleep and energy expenditure are daily means of a 1-week measurement period with the actigraphy device. J, Joule; BMI, body mass index; BMI_z_, age and sex corrected BMI z-scores; where $BMI_{z}=\;\left[{\left(\frac{BMI}{M}\right)}^{L}-\text{1}\right]÷\left(L\times S\right)\;$and M, L, and S are age and sex dependent parameters obtained from Centers for disease control and prevention (www.cdc.gov)*.

### MRI

All images were acquired with an Ingenia 3T (Philips Healthcare, Best, The Netherlands) using the WholeBody dStream coil array. For body-composition analysis, a set of up to 10 overlapping axial image stacks of in and opposite phase images with reconstruction of both real and imaginary components. Common parameter settings of the spoiled gradient echo sequence used in the body-composition protocol were flip angle 10°, echo times (TEs) 1.14/2.30 ms, field of view (FOV) 560 × 385 mm^2^, scan matrix 320 × 220, slice thickness 1.75, 30 mm overlap between stacks. The whole body was covered beginning at the head with an image stack of 149 slices, repetition time (TR) 3.8 ms, and a sense factor of 2.5, followed by 6 breath hold stacks with 85 slices, TR 2.8 ms, and sense factor 2.5. Finally, the legs were covered by three 150 slice stacks, TR 6.0 ms, and a sense factor of 4. Water-fat images were reconstructed by phase-sensitive reconstruction(34). For sBAT analysis 6-echo 3D spoiled gradient echo images were collected over the neck, flip angle 10°, TEs 1.14/2.29/3.44/4.59/5.74/6.89 ms, TR 26 ms, FOV 380 × 303 mm^2^, scan matrix 216 × 218, slice thickness 1.75 mm, 69 slices, and a sense factor of 2. Based on the first two echoes, all image stacks were water-fat separated using phase sensitive reconstruction (34). For the liver and neck images the T2^*^ and lipid spectral effects were corrected for using the magnitude-based chemical shift technique (35) with a 6-peak (36) model using the two-echo water-fat separation for initialization. The water-fat images were separated using Matlab R2015b (The MathWorks, Inc., Natick, Massachusetts, United States).

### Image Analysis

**Figure 1:**
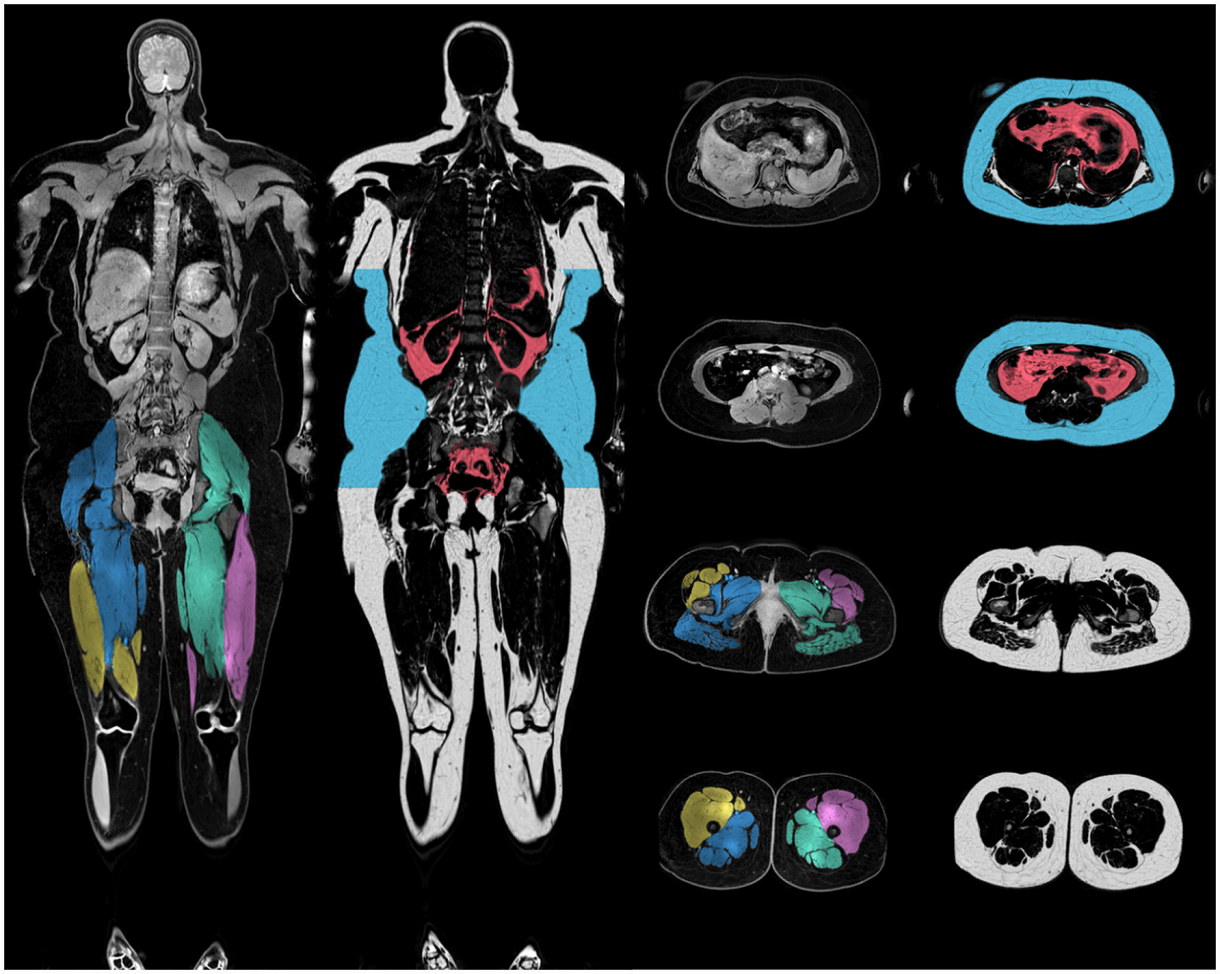
Illustration of body-composition. Merged and calibrated water-fat MRI images. The anterior and posterior thigh definitions are shown in the water image and visceral and subcutaneous adipose tissue definitions in the fat images. Intra muscular adipose tissue was defined as the average fat fraction of the anterior thighs.

**Figure 2:**
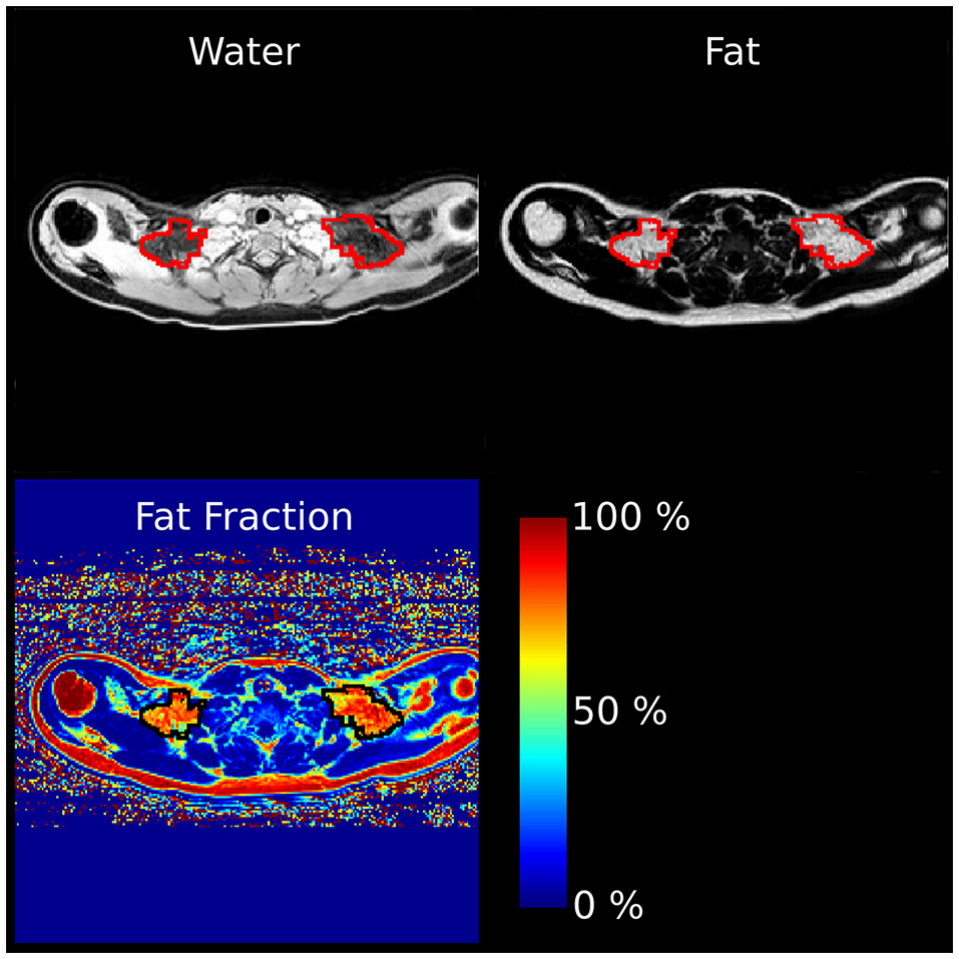
Representative sBAT segmentation. The axial water, fat and fat fraction images shows a cross section of the supraclavicular BAT segmentation. The area circled in red (fat and water images) or black (fat fraction image) was defined as sBAT.

For the body-composition analysis, the whole-body water-fat images were calibrated using adipose tissue as an intensity reference (37, 38) and merged into water-fat images with whole-body coverage. Based on the whole-body images the VAT volume, ASAT, and thigh muscle volume were automatically measured, and quality controlled by a trained operator, using AMRA Profiler Research (AMRA Medical AB, Linköping, Sweden) (Figure 1). Visceral adipose tissue was defined as adipose tissue within the abdominal cavity, excluding adipose tissue outside the abdominal skeletal muscles, and adipose tissue and lipids within and posterior of the spine and posterior of the back muscles; ASAT was defined as subcutaneous tissue in the abdomen from the top of the femoral head to the top of the thoracic vertebrae T9; and, the thigh definition comprised the gluteus, iliac, adductor, hamstring muscles, quadriceps femurs, and sartorius. Adipose tissue volume was given by integration of the calibrated fat image (37), and fat free muscle tissue volume was given by voxels within the thigh segmentation with < 50% adipose tissue (39). Muscle fat infiltration (MFI) was defined as the concentration of adipose tissue within the quadriceps femurs and sartorius after removing voxels with more than 50% adipose tissue. The fat fraction (fat/[fat + water]) was measured within the supraclavicular compartment and used as an indicator of BAT volume, since the fat fraction has been shown to correlate with BAT in both rodent (40, 41) and human (42, 43). The sBAT compartment was segmented using multi-atlas segmentation implemented in Matlab as previously described by Romu et al. (44) (Figure 2).

### Statistics

In this study, the estimated fat volumes VAT, ASAT, and TAAT were normalized by height squared to account for differences in body height, giving the variables TAATi, VATi, and ASATi with index “i” to denote that variables represent the height adjusted index version of the variable. A visceral adipose tissue ratio (VATR) was also calculated, where VATR = VAT/(VAT + ASAT). We used single variable between-group tests to determine whether there were any group differences for any of the adipose tissue measures. Age, gender, sleep, and total energy expenditure were entered as covariates for all single variable tests. We found no correlations between medication and any of the adipose tissue measures. To further rule out the effect of medication, we tested the dependency of each of the adipose tissue measures to two groups of medication: Selective serotonin reuptake inhibitor (SSRI) and stimulants, in a one-way ANOVA. We found that adipose tissue measures were unrelated to any of the prescribed medications. Medication was therefore not entered as a covariate for any of the subsequent tests.

## Results

### No Difference in Brown Adipose Tissue Fat Fraction Between Narcolepsy Patients and Healthy Controls

**Figure 3:**
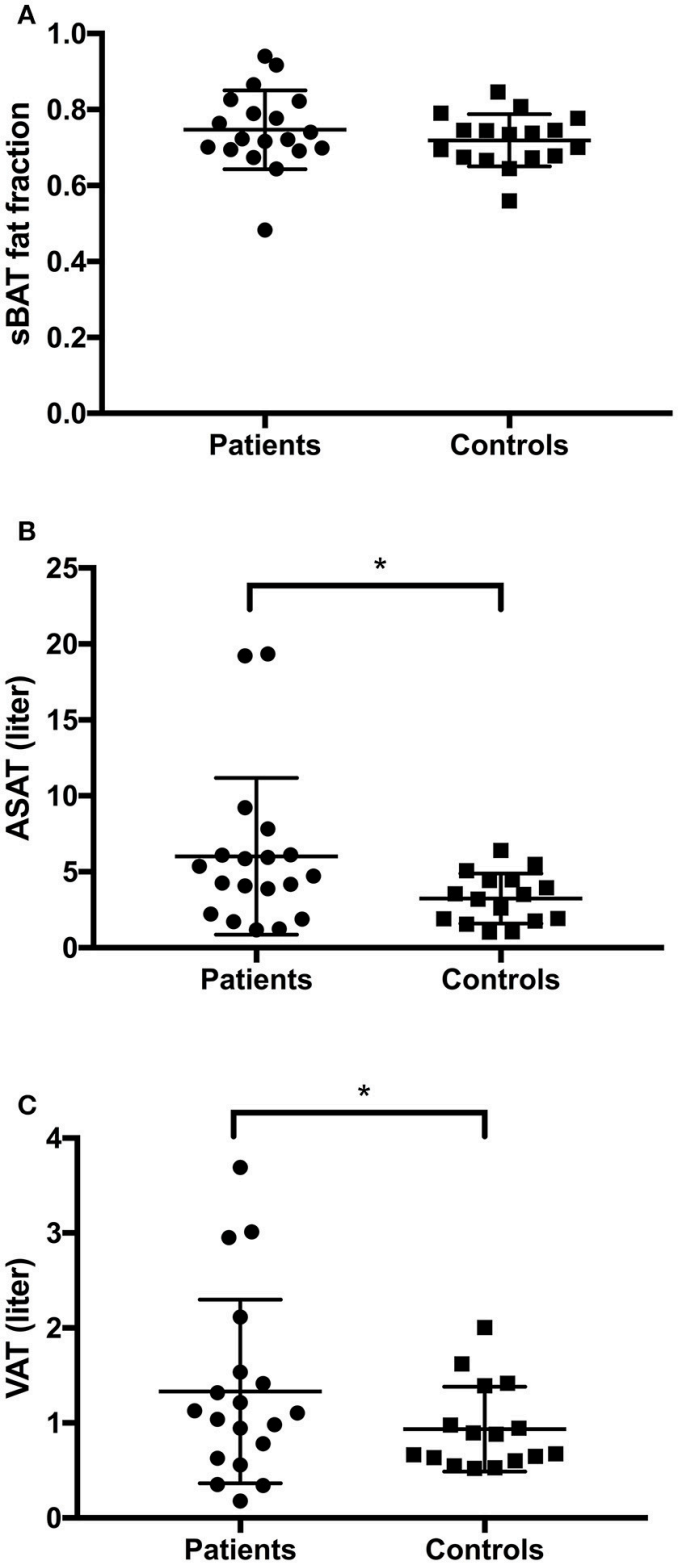
Results adipose tissue measures. **(A)** No difference in sBAT fat fraction between narcolepsy patients and healthy controls (*p* = 0.207). **(B)** Narcolepsy patients had more subcutaneous abdominal adipose tissue (ASAT). **(C)** Narcolepsy patients have more visceral adipose tissue (VAT) than controls. sBAT, supraclavicular brown adipose tissue; ASAT, abdominal subcutaneous adipose tissue; VAT, Visceral adipose tissue. ^*^p < 0.05.

We found no significant group differences in sBAT fat fraction, an indirect indicator of BAT volume, when comparing between patients (0.75, SD 0.104) and controls (0.72, SD 0.069) (*p* = 0.207) (Table 3; Figure 3A).

**Table 3:** Results of adipose tissue measures.

	**Narcolepsy patients**	**Controls**	**Statistics**
sBAT (fat fraction)	0.746 SD 0.104	0.719 SD 0.069	*p* = 0.207
ASAT (liters)	mean 6.019 SD 5.159	3.244 SD 1.646	*p* = 0.003
TAAT	mean 7.350 SD 6.058	4.179 SD 1.998	*p* = 0.003
TAATi	mean 2.592 SD 2.201	1.354 SD 0.738	*p* = 0.003
TAATR	0.406 SD 0.213	0.367 SD 0.261	*p* = 0.739
VAT (liters)	mean 1.331 SD 0.966	0.935 SD 0.935	*p* = 0.012
VATi	0.468 SD 0.338	0.297 SD 0.146	*p* = 0.006
VATR	0.192 SD 0.054	0.239 SD 0.068	*p* = 0.019
MFI	0.044 SD 0.011	0.043 SD 0.009	*p* = 0.934
Muscle ratio	0.148 SD 0.047	0.141 SD 0.069	*p* = 0.255

*Table shows mean values for all collected adipose tissue measures. sBAT, supraclavicular brown adipose tissue; ASAT, abdominal subcutaneous adipose tissue; TAAT, total abdominal adipose tissue (VAT+ASAT); TAATi, total abdominal adipose tissue index (TAAT/ height^2^); TAATR, total abdominal adipose tissue ratio (VAT+ASAT)/(ASAT+VAT+thigh muscle); VAT, visceral adipose tissue; VATi, Visceral adipose tissue index (VAT/ height^2^); VATR, VAT ratio (VAT/VAT+ASAT); MFI, muscle fat infiltration; Muscle ratio, thigh muscle/body weight*.

### Adolescent Narcolepsy Patients Have More Abdominal Adipose Tissue Than Healthy Controls

We found that total abdominal adipose tissue (TAAT) was higher in the narcolepsy patients compared to controls, *p* = 0.003 (Table 3), as well as BMI, *p* = 0.039, and age and sex corrected BMI z-scores BMI_z_, *p* = 0.004 (Table 2). We also found that ASAT, a measure of subcutaneous tissue in the abdomen from the top of the femoral head to the top of the thoracic vertebrae T9 was higher in narcolepsy patients *p* = 0.003 (Table 3; Figure 3B). VAT is a measure of total visceral abdominal adipose tissue and we found that adolescent narcolepsy patients have a larger total volume of VAT, *p* = 0.012 (Table 3; Figure 3C). We normalized to account for height and found that the patients had a higher TAATi, *p* = 0.003 compared to healthy controls (Table 3). We also found the same group differences when comparing VATi, *p* = 0.006 (Table 3). These results were all significant even after correcting for age, gender, sleep and total energy expenditure. We found no significant group differences for MFI (*p* = 0.934) or muscle ratio (*p* = 0.255), as well as no significant group differences for TAATR (*p* = 0.7) (Table 3).

### The Visceral Adipose Tissue-to-Total Abdominal-Adipose Tissue Ratio Is Lower in Narcolepsy Patients

**Figure 4:**
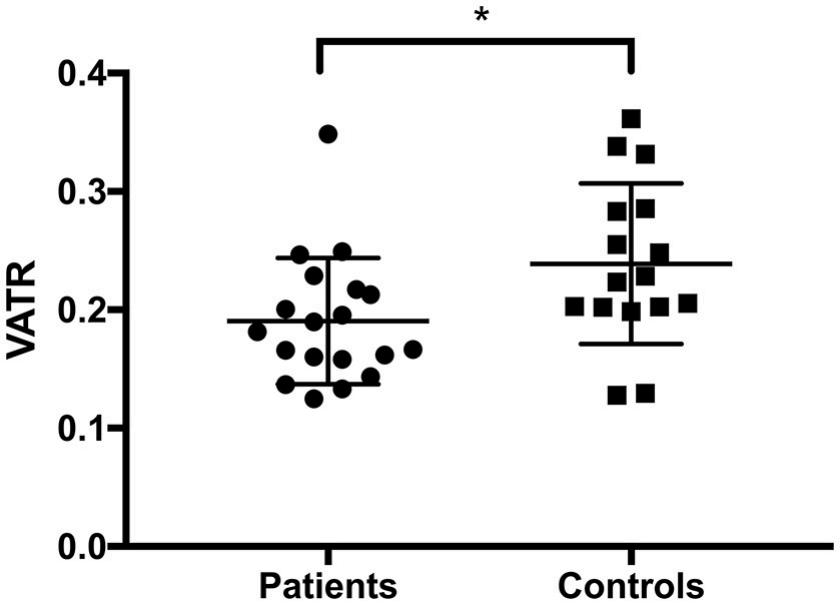
Visceral adipose tissue ratio. We found a lower ratio between VAT and VAT+ASAT (VATR) in narcolepsy patients. VAT, Visceral adipose tissue; VATR, visceral adipose tissue ratio, VAT/VAT+ASAT; ASAT, abdominal subcutaneous adipose tissue. ^*^p < 0.05.

When comparing VAT normalized to TAAT i.e., VATR, we found that the narcolepsy patients had a lower VATR than the healthy controls, *p* = 0.019 (Table 3; Figure 4). Corrections were made for age, sleep, gender, and total energy expenditure.

## Discussion

The aim of this paper was to investigate body fat distribution in adolescent narcolepsy patients, specifically sBAT fat fraction as narcolepsy patients have very low levels of orexin, a hormone shown to regulate BAT. Our main findings were three-fold and can be summarized as follows.

- No group differences were found in sBAT fat fraction.
- Narcolepsy patients had more TAAT, matching a central obesity phenotype. We also observed higher volumes of ASAT as well as VAT, even after normalizing to height.
- Even though narcolepsy patients had more total VAT, we unexpectedly found a lower VATR indicating a relative increase in ASAT.

### No Group Differences in Supraclavicular BAT Fat Fraction

We wanted to explore if reduced levels of orexin, a primary indicator of narcolepsy type 1, could have an effect on BAT volume, and we hypothesized that we would see differences in sBAT fat fraction, an indicator of BAT volume, between our patients and controls. Murine models have demonstrated a critical role for orexin in the regulation of BAT, specifically showing that a reduction or lack of orexin in neonate rodents inhibits the ability of brown preadipocytes to correctly differentiate (25, 26). This in turn could contribute to an impaired thermogenesis, which may later induce obesity (25, 45, 46). While we did not observe any difference in sBAT fat fraction between the narcolepsy patients and controls, it is important to keep in mind that the failure of the thermogenic mechanism, as it is described in rodents, is seated in a defective BAT development due to embryonic loss of orexin. In most humans, symptoms of narcolepsy typically don't appear until adolescence (1) and even though our patient group consisted of adolescents, it is possible that the developmentally sensitive time period for BAT lies even earlier. Importantly, there may also be changes related to BAT function, such as to its activation or morphology that cannot be measured using the methods within this study.

While an increase to the metabolic rate of BAT in humans has been demonstrated following both cold exposure as well as after a meal (44, 47–51), orexin has only been directly implicated in the activation of BAT in animal models (52). Additionally a recent study using an *in-vitro* cell model of human brown and white adipocytes found no increase in BAT activation following orexin treatment (53). A limitation to this study is that we measured BAT in room temperature under unstimulated conditions. Additionally, we did not measure orexin at the time of the study, however we were able to extract orexin measures from the clinical records of nine of the patients (Table 1). We found no significant group differences in sBAT fat fraction when comparing the patients with known orexin concentrations to the healthy controls, suggesting that orexin has no effect at all on BAT, at least under thermoneutral conditions.

### Abdominal Adipose Tissue Distribution in Adolescent Narcolepsy Patients

Previous research has shown that narcolepsy patients have a higher BMI and are more likely to be obese, with a larger waist circumference compared to their healthy peers (3, 4, 10, 54). In support of previous research, we found that the narcolepsy patients had a higher BMI, with more TAAT than the healthy controls, including more total ASAT and VAT, but with no differences in MFI or muscle ratio. Abdominal adiposity is one of the hallmarks of central obesity (7, 55) and high VAT has been linked to an increased risk for diabetes type 2, cardiovascular disease, metabolic syndrome, and even depression (56, 57). Additionally, previous research shows that narcolepsy patients may be more insulin sensitive with a lower rate of lipolysis, which suggests an increased risk for narcolepsy patients in developing metabolic syndrome (58).

Unexpectedly, we found that the relative VAT volume; VATR was significantly lower indicating a relative increase in ASAT. VATR has previously been found to be superior to VAT volume as a measure of VAT risk (59–61) and is typically measured by comparing the ratio of visceral to subcutaneous adipose tissue, either from the thigh (62) or as presented in this paper, the abdomen (VATR) (63). A clinical study by Porter et al. compared the metabolic and cardiovascular disease risk between people with either high or low VAT in conjunction with high or low volumes of subcutaneous adipose tissue. They found that disease risk for those who were in the highest percentile for VAT was inversely related to the volume of subcutaneous adipose tissue, while for those in the lower percentiles, this relationship was not seen. They concluded that subcutaneous adipose tissue might potentially have a protective effect in some individuals, specifically in those with a higher volume of VAT (64). Other research shows that removal of subcutaneous tissue through liposuction had no long-term effect on metabolic risk factors for coronary heart disease in obese women (65) and in one study even worsened postprandial blood lipid concentrations (66). These findings are supported and extended by research on murine models, where surgical removal of subcutaneous adipose tissue from high VAT animals caused a decrease in glucose tolerance and insulin sensitivity (67, 68). Reversely, transplanting of subcutaneous tissue into high VAT animals resulted in alleviation of diet induced metabolic disease (67, 69, 70). We might speculate that the increase in subcutaneous adipose tissue observed in narcolepsy patients is in response to an excess of fatty acids in the vascular compartment. Future studies should address the existence and eventual protective function of increased VATR in narcolepsy.

It is well-established that long-term use of psychotropic drugs can affect weight management. We therefore tested dependency of both antidepressants and stimulants to any of the adipose tissue measures in our narcolepsy patients, and found no significant relationships. These findings are supported by a study from 2004, which reported no significant difference in BMI between pediatric narcolepsy patients who had received prior treatment with psychotropic drugs and those who had not (10).

## Conclusion

We conclude that adolescent narcolepsy patients gain more weight than their healthy peers and that they have more TAAT, with no difference in relative muscle or in muscle fat content. We also conclude that the total VAT volume was higher in narcolepsy patients, corresponding to central obesity and possibly putting them at an increased risk for metabolic syndrome. Unexpectedly, we also found that the increase in subcutaneous abdominal adipose tissue (ASAT) may not be accompanied by a relative increase in VAT, as we found that VATR was lower in the narcolepsy patients. Additionally, contrary to our expectations there were no differences in sBAT fat fraction between patients and controls, suggesting that orexin may have no effect at all on BAT, at least under thermoneutral conditions.

## Ethics Statement

The study was performed in accordance with the Helsinki Declaration and approved by the Regional Ethical Review Board in Linköping, Sweden (2013/99-31). All participants gave written informed consent to participate.

## Author Contributions

NM, AS, TH,ND, A-ML, MB, OD, and ME: conception and design of the work. NM, TR: data acquisition and analysis. NM, TR, OD, and ME: interpretation of data. NM: drafting of the manuscript. All authors revised the manuscript critically for important intellectual content and approved the final version of this manuscript.

### Conflict of Interest Statement

TR, MB, and OD are employees and shareholders of AMRA. The remaining authors declare that the research was conducted in the absence of any commercial or financial relationships that could be construed as a potential conflict of interest.
